# Profiling regulatory T lymphocytes within the tumor microenvironment of breast cancer via radiomics

**DOI:** 10.1002/cam4.6757

**Published:** 2023-12-11

**Authors:** Wenying Jiang, Ruoxi Wu, Tao Yang, Shengnan Yu, Wei Xing

**Affiliations:** ^1^ Department of Radiology The Third Affiliated Hospital of Soochow University Changzhou China; ^2^ Department of Breast Surgery The Third Affiliated Hospital of Soochow University Changzhou China; ^3^ Department of Breast Surgery Gansu Provincial Maternity and Child Care Hospital Lanzhou China

**Keywords:** breast cancer, landmark analysis, radiomics, regulatory T lymphocytes, tumor microenvironment

## Abstract

**Objective:**

To generate an image‐driven biomarker (Rad_score) to predict tumor‐infiltrating regulatory T lymphocytes (Treg) in breast cancer (BC).

**Methods:**

Overall, 928 BC patients were enrolled from the Cancer Genome Atlas (TCGA) for survival analysis; MRI (*n* = 71 and *n* = 30 in the training and validation sets, respectively) from the Cancer Imaging Archive (TCIA) were retrieved and subjected to repeat least absolute shrinkage and selection operator for feature reduction. The radiomic scores (rad_score) for Treg infiltration estimation were calculated via support vector machine (SVM) and logistic regression (LR) algorithms, and validated on the remaining patients.

**Results:**

Landmark analysis indicated Treg infiltration was a risk factor for BC patients in the first 5 years and after 10 years of diagnosis (*p* = 0.007 and 0.018, respectively). Altogether, 108 radiomic features were extracted from MRI images, 4 of which remained for model construction. Areas under curves (AUCs) of the SVM model were 0.744 (95% CI 0.622–0.867) and 0.733 (95% CI 0.535–0.931) for training and validation sets, respectively, while for the LR model, AUCs were 0.771 (95% CI 0.657–0.885) and 0.724 (95% CI 0.522–0.926). The calibration curves indicated good agreement between prediction and true value (*p* > 0.05), and DCA shows the high clinical utility of the radiomic model. Rad_score was significantly correlated with immune inhibitory genes like CTLA4 and PDCD1.

**Conclusions:**

High Treg infiltration is a risk factor for patients with BC. The Rad_score formulated on radiomic features is a novel tool to predict Treg abundance in the tumor microenvironment.

## Graphical abstract

The risk exert by Treg infiltration on breast cancer patients was confirmed via landmark analysis. Then, a radiomic score to estimate Treg abundance in the tumor microenvironment was formulated, which was found to be correlated to inhibitory immune genes.

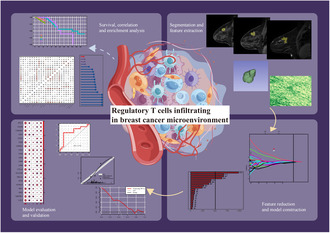

## 1 INTRODUCTION

While much work has been done, breast cancer (BC) ranks as the major cause of cancer‐related mortality.^1^ About 30% of early‐stage patients would eventually become metastatic or resistant to therapies, hence, there is still much to do.^2^ It is urgent to learn about interactions between immunocytes and BC developments and their potential prognostic and predictive capabilities.^3^

Foxp3^+^ regulatory T lymphocytes (Treg) are identified as the dominant mechanism of immune escape from tumor‐specific effector T lymphocyte responses.^4^ They are regarded as a major obstacle to effective cancer immunotherapy, hence targeting Treg could be a promising approach for BC immunotherapy, such as immune checkpoint inhibitors (ICI).^5^ Treg infiltrating reflects real‐time anti‐tumor immune response and helps oncologists to assess patients' prognosis.^6^ Yet, biopsies and pathologic reviews are incapable to reflect tumor heterogeneity, due to sampling bias.

In addition to morphology, macroscopic imaging systems also give us molecular and functional information about the tumor.^7^ High‐dimension radiomics is now considered a novel biomarker discovery platform.^8^ Association analysis between MRI features and BC molecular, genomic, and related characteristics is increasing, constantly.^9^ In vivo, imaging is noninvasive and can be performed at multiple time points.

In this study, we aimed to develop a non‐invasive and cost‐effective approach that can be repeatedly measured to inform about Tregs infiltration in BC patients, in addition to the prognostic value of Treg in BC.

## 2 MATERIALS AND METHODS

### 2.1 Survival and comparison analysis of Treg infiltration in BC

**FIGURE 1:**
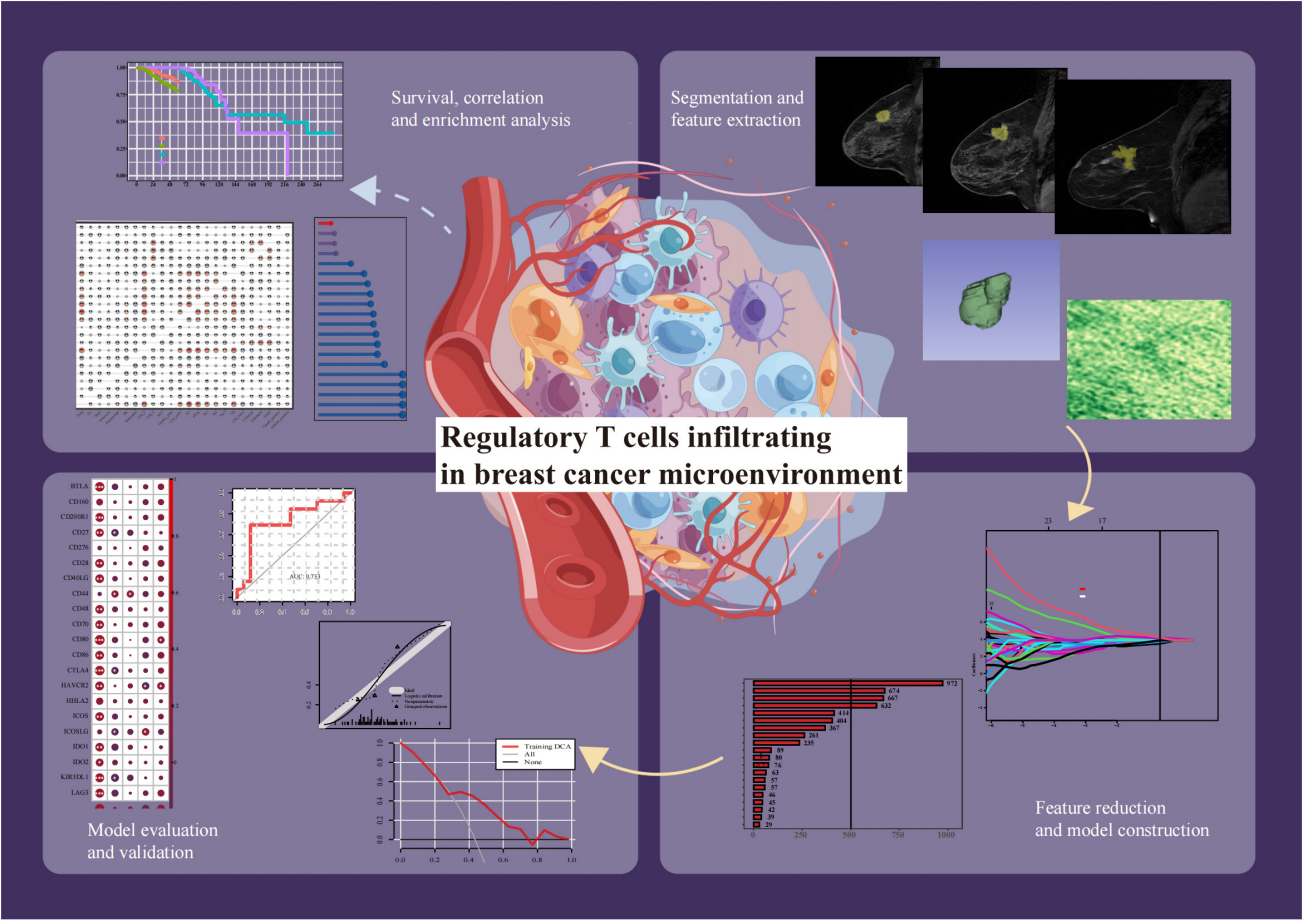
Flow chart of the study.

RNA sequencing data (*n* = 1097) of invasive breast carcinoma (BRCA) were acquired from the Cancer Genome Atlas (TCGA, https://portal.gdc.cancer.gov/). The flow diagram is shown in Figure 1. The inclusion criteria for survival and correlation analysis of Treg were as follows: (1) primary and treatment‐naive BC patients (*n* = 1082), (2) survive over 30 days after diagnosis (*n* = 1032), (3) female patients (*n* = 1020), while the exclusion criteria were: (1) main tumor cannot be measured (Tx stage, *n* = 1017), (2) missing important clinical information, such as age, TNM stages and chemoradiotherapy (*n* = 968), (3) unavailable RNA sequencing data (*n* = 928).

Among these TCGA‐BRCA patients, 130 have MR images stored in the Cancer Imaging Archive (TCIA, https://www.cancerimagingarchive.net); in which all images must be submitted following a standard deidentification pass compliant with the DICOM Standard. MRI scans of BC patients from the intersection of TCIA and TCGA datasets were employed for the construction of the radiomic model to predict Treg infiltration level (*n* = 130). MRI scans were performed on 1.5‐T GE Medical Systems or Siemens MRI scanners. The median pixel spacing, slice thickness, and spacing between slices were 0.7, 2.2, and 2.2 mm, respectively. The median repetition time and echo time were 7.4 milliseconds (ms) and 3.6 ms. The exclusion for radiomic analysis were images of post‐surgery or poor quality (*n* = 29) The final number of cases remaining for radiomic study is 101.

The gene expression matrix of BC was uploaded to the ImmuCellAI database (http://bioinfo.life.hust.edu.cn/ImmuCellAI#!/), and Treg infiltration was calculated for each sample. Patients were dichotomized into Treg^high^ and Treg^low^ groups for survival analysis at a cutoff calculated via the R package “survminer”, The surv_cutpoint() function, which used the maximally selected rank statistics, was used to split each of the samples into high‐ or low‐infiltration groups (Available online at: https://CRAN.R‐project.org/package=survminer). Kaplan–Meier curves depicted the differences in overall survival between the high and low Treg infiltration groups and followed by landmark analysis with 5 and 10 years after BC diagnosis as the landmarks. Then the association of Treg with OS in BC patients was analyzed via the COX proportional risk model.

Comprehensive analysis of correlations between Treg infiltration and clinical parameters, abundance of other immune cells, and immune genes were calculated by Spearman's rank correlation coefficient, based on the ImmuCellAI database and published literature.^10^ Gene set enrichment analysis (GSEA) of differentially expressed genes (DEG) between Treg^high^ and Treg^low^ groups was performed on Hallmark (h.all.v7.5.1.symbols.gmt) and KEGG (c2.cp.kegg.v7.5.1.symbols.gm) gene sets. Mutation information in the maf format of TCGA‐BRCA was downloaded for comparison between high and low Treg infiltration groups. Wilcox test was used to analyze the significance of the difference in tumor mutation load (TMB) between high and low Treg groups.

### 2.2 Radiomic signature to predict Treg abundances in BC

To reduce the discrepancy of parameters employed in different MRI scanners, image preprocessing was applied. A bias field correction using the N4ITK algorithm was to correct for potential effects due to the inhomogeneity of the magnetic field. MRI intensity inhomogeneity was reduced by spatial resampling to 1 × 1 × 1 mm and image intensity normalization. A professional imaging physician (AA, 10 years of experience in breast MRI) combined plain scan and dynamic enhancement MRI images for a comprehensive evaluation to determine the location of the lesion by adjusting the appropriate window width and window level and bilaterally comparing it to see if there were abnormal soft tissue masses, or areas of structural changes, signal differences, and abnormal enhancement to identify the tumor area. The most obvious phase of MRI lesion post‐contrast was selected, and the entire tumor area was outlined using 3D Slicer software (version 4.10.2) to extract high‐dimensional imaging histological features. The “random number table method” selected 30 samples to be outlined by another physician (BB, 3 years of experience in breast imaging). Intraclass correlation efficiency (ICC) was calculated, and features with ICC ≥0.75 were selected for feature screening.

The radiomic cohort was randomly split into training and validation sets at the ratio of 7:3. The radiomic features of the training set were normalized; those in the validation set were normalized accordingly. Repeat least absolute shrinkage and selection operator (LASSO) filtered the features and features with a frequency of more than 500 occurrences in 1000 screening sessions were selected for the prediction model of Treg infiltration. At first, retained features were modeled via a support vector machine (SVM) algorithm, and then the logistic regression (LR) algorithm was applied for comparison.

The prediction performance was assessed by the area under the receiver operating characteristic (ROC) and precision‐recall (PR) curves (AUCs), accuracy (ACC), sensitivity (SEN), specificity (SPE), positive predictive value (PPV), negative predictive value (NPV), calibration curve, Brier score and decision curve analysis (DCA). The radiomic scores (rad_score) for Treg infiltration estimation were calculated for comparison between groups with high and low Treg infiltration.

Finally, correlation analysis between Rad_score and immune inhibitory molecules was conducted to explore the underlying mechanism of the radiomic model.

### 2.3 Statistics

R software was used for statistical analysis. Statistical differences between the two groups were determined by Wilcoxon rank‐sum tests. Log‐rank test was to test the difference in survival rate. The Delong test was to compare the AUCs of ROC and PR curves. The Hosmer–Lemeshow test was to test the goodness of fit of the prediction model. A two‐sided *p* value <0.05 was considered statistically significant.

## 3 RESULTS

### 3.1 Survival and comparison analysis of Treg infiltration in BC

**FIGURE 2:**
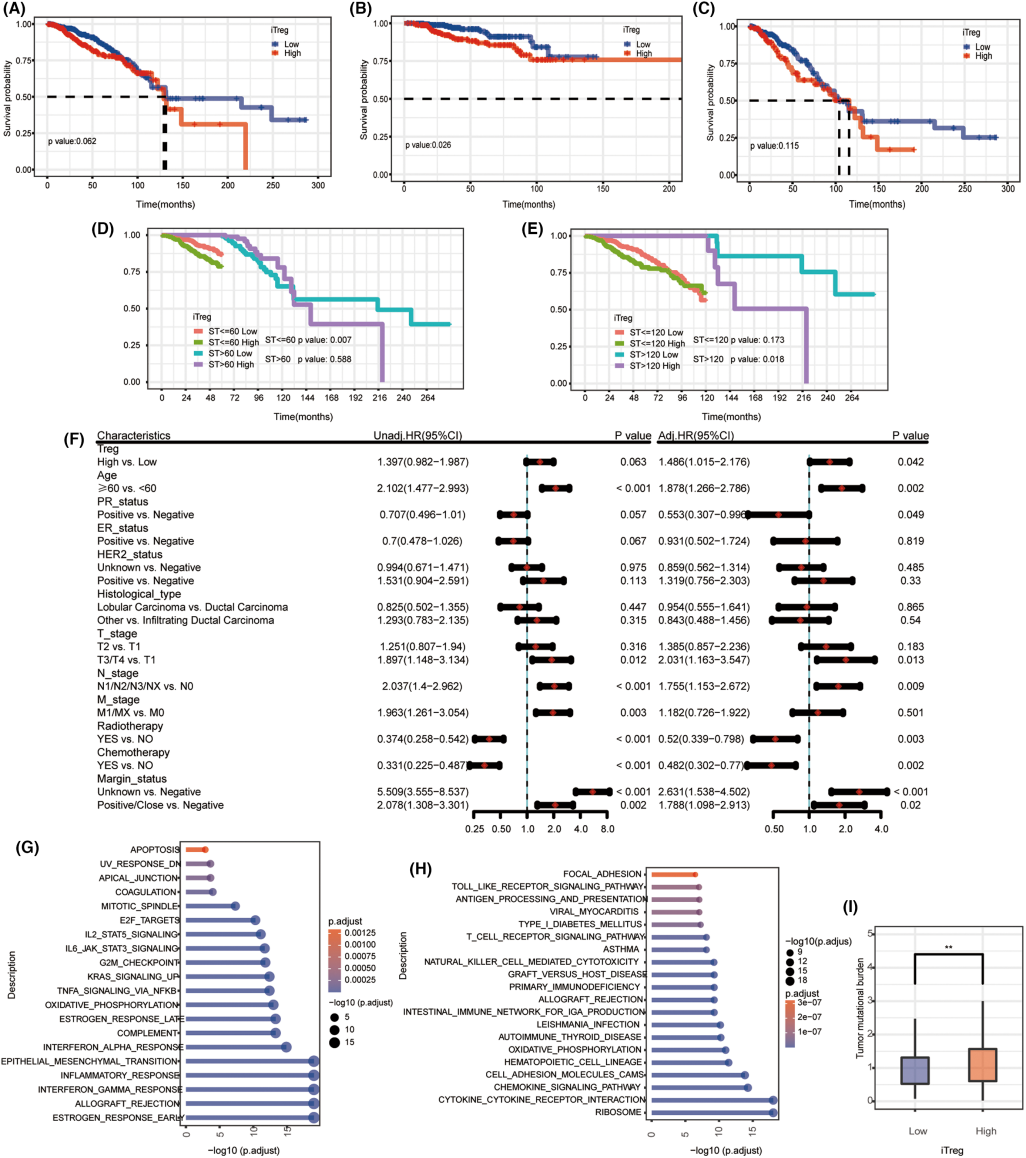
Survival and comparison analysis of regulatory T lymphocytes (Treg) infiltration in breast cancer: Kaplan–Meier curves of (A) the whole cohort, (B) the chemotherapy sub‐cohort and (C) the sub‐cohort without chemotherapy; landmark analysis with (D) 5 years and (E) 10 years after breast cancer diagnosis as the landmarks; (F) the Forrest plot of COX proportional risk model; gene set enrichment analysis of differentially expressed genes between high and low Treg infiltrating groups performed on (G) Hallmark and (H) KEGG gene sets; (I) comparison of tumor mutation burden between high and low Treg infiltrating groups.

Finally, 928 BC cases were included in the survival analysis. and the clinical information is shown in Table 1. There were significant differences in the distributions of age, hormonal receptor status, histology, and chemotherapy between the high and low Treg infiltration groups (*p* < 0.05). Kaplan–Meier curves showed that higher Treg infiltration was associated with worse overall survival (OS) in most of the curves (*p* = 0.062, Figure 2A), but they were intertwined. In the sub‐cohort with chemotherapy, higher Treg infiltration was associated with worse OS (*p* = 0.026, Figure 2B), but Kaplan–Meier curves were also intertwined in the sub‐cohort without chemotherapy(*p* = 0.115, Figure 2C). Further analysis with landmarks demonstrated more details: Treg infiltration was a risk factor for BC patients in the first 5 years and after 10 years of diagnosis (*p* = 0.007 and 0.018, Figure 2D,E, respectively). High Treg was a risk factor for OS in the univariate Cox regression analysis (HR = 1.397, 95% CI 0.982–1.987); after adjustment, Treg infiltration was an independent prognostic factor for BC patients (HR = 1.486, 95% CI 1.015–2.176, Figure 2F).

**TABLE 1:** Baseline characteristics of patients included in the survival analysis.

Variables	Total (*n* = 928)	Low (*n* = 569)	High (*n* = 359)	*p*
Age, *n* (%)				
<60	506 (55)	292 (51)	214 (60)	0.016
≥60	422 (45)	277 (49)	145 (40)	
PR_status, *n* (%)				
Negative	298 (32)	142 (25)	156 (43)	<0.001
Positive	630 (68)	427 (75)	203 (57)	
ER_status, *n* (%)				
Negative	210 (23)	84 (15)	126 (35)	<0.001
Positive	718 (77)	485 (85)	233 (65)	
HER2_status, *n* (%)				
Negative	493 (53)	305 (54)	188 (52)	0.229
Unknown	295 (32)	187 (33)	108 (30)	
Positive	140 (15)	77 (14)	63 (18)	
Histological_type, *n* (%)				
Invasive ductal carcinoma	655 (71)	378 (66)	277 (77)	0.002
Invasive lobular carcinoma	185 (20)	131 (23)	54 (15)	
Other	88 (9)	60 (11)	28 (8)	
T_stage, *n* (%)				
T1	250 (27)	150 (26)	100 (28)	0.13
T2	528 (57)	316 (56)	212 (59)	
T3/T4	150 (16)	103 (18)	47 (13)	
N_stage, *n* (%)				
N0	430 (46)	267 (47)	163 (45)	0.7
N1/N2/N3/NX	498 (54)	302 (53)	196 (55)	
M_stage, *n* (%)				
M0	771 (83)	469 (82)	302 (84)	0.561
M1/MX	157 (17)	100 (18)	57 (16)	
Radiotherapy, *n* (%)				
NO	442 (48)	273 (48)	169 (47)	0.841
YES	486 (52)	296 (52)	190 (53)	
Chemotherapy, *n* (%)				
NO	406 (44)	270 (47)	136 (38)	0.005
YES	522 (56)	299 (53)	223 (62)	
Margin_status, *n* (%)				
Negative	778 (84)	481 (85)	297 (83)	0.728
Unknown	51 (5)	29 (5)	22 (6)	
Positive/close	99 (11)	59 (10)	40 (11)	

Treg infiltration was found to be significantly correlated to patients' age, estrogen receptor status, progesterone receptor status, histological type, and chemotherapy; as well as various immune cell abundance and gene expression (Figures S1–, S3).DEG between Treg^high^ and Treg^low^ groups were significantly enriched in estrogen and interferon‐gamma response, and IL6_JAK_STAT3 and IL2_STAT5 signaling in the Hallmark gene set (Figure 2F); primary immunodeficiency, T cell, B cell receptor signaling, leukocyte transendothelial migration, et al. in the KEGG gene set (Figure 2G). Also, tumor mutation burden (TMB) was higher in the high Treg infiltration group (*p* < 0.01, Figure 2H).

### 3.2 Radiomic signature to predict Treg abundances in BC

**FIGURE 3:**
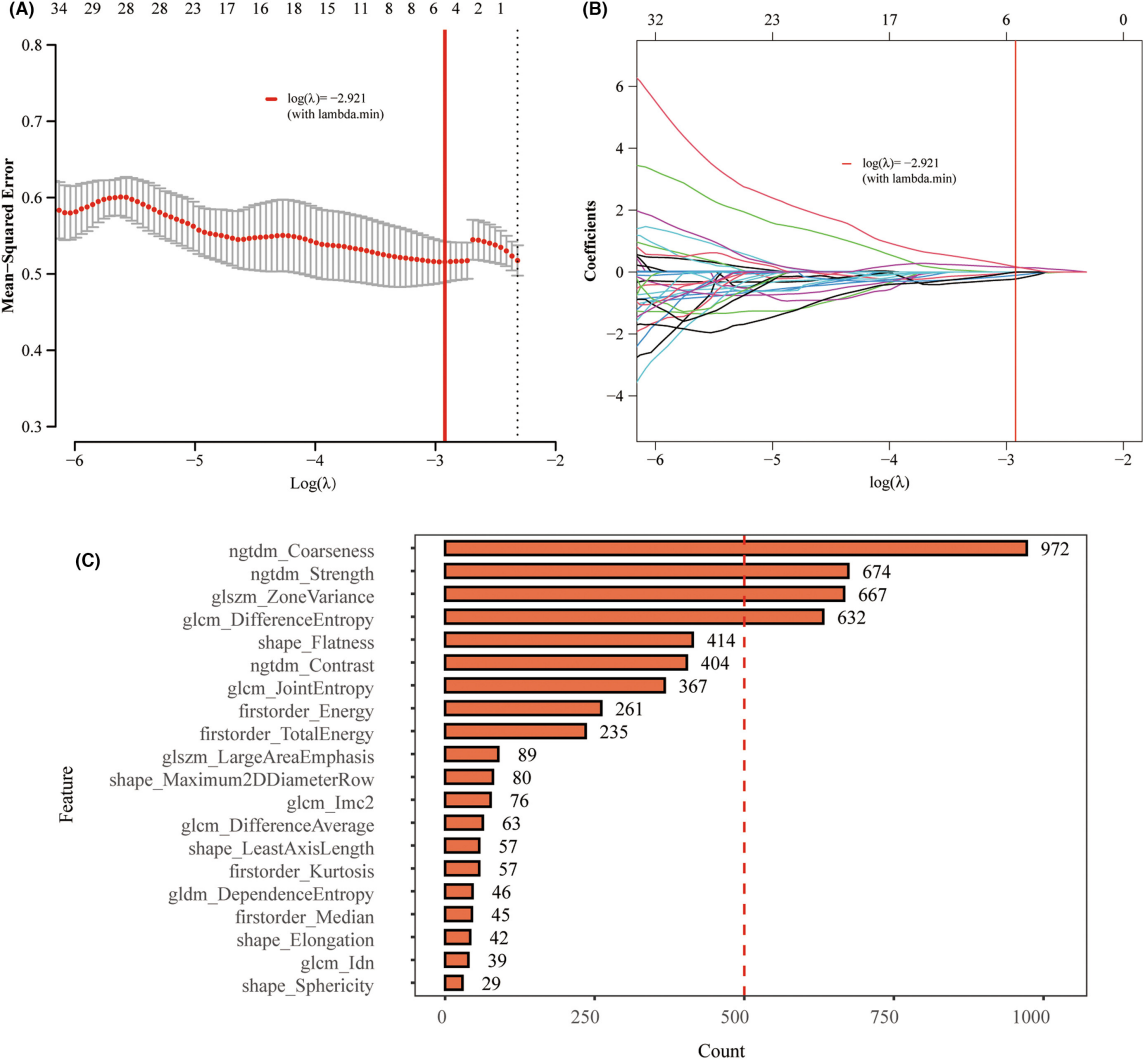
Radiomic feature selection using the repeat least absolute shrinkage and selection operator (LASSO). (A) The selection of tuning parameter (*λ*) in the LASSO model via minimum criteria. The area under the curve (AUCs) was plotted versus log (*λ*). Dotted vertical lines were drawn at the optimal value by using the minimum criteria and the 1 standard error of the minimum criteria (the 1 − standard error criteria). (B) LASSO coefficient profiles of selected features. A vertical line was plotted at the optimal *λ* value to select features with non‐zero coefficients. (C) Four features with frequencies over 500 in Repeat LASSO screening remained for the next model construction.

**FIGURE 4:**
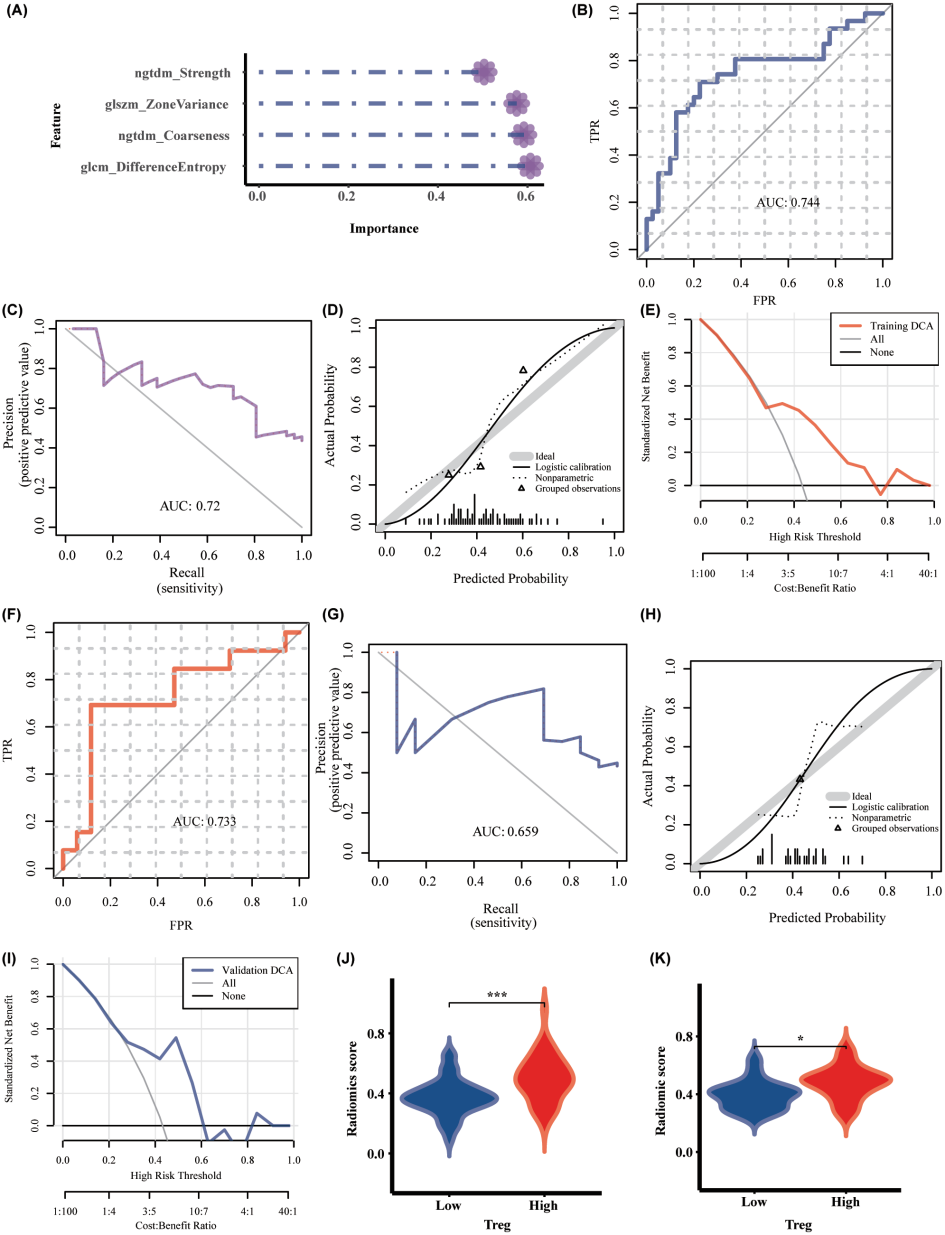
The importance of features and evaluation of the radiomic model based on support vector machine algorithm: (A) the importance of features; (B) the ROC curve, (C) the PR curve, (D) the calibration curve, and (E) DCA in the training set; (F) the ROC curve, (G) the PR curve, (H) the calibration curve and (I) DCA in the validation set; comparison of Rad_score between high and low regulatory T lymphocyte infiltrating groups in the (J) training and (K) validation sets. DCA, decision curve analysis; PR, precision‐recall; Rad_score, radiomic score; ROC, receiver operating characteristic.

Altogether, 108 radiomic features were extracted from MRI images, 92.5% of which (99 features) with ICC ≥0.75 were retained for further analysis. Four features with frequencies over 500 in Repeat LASSO screening remained for model construction (Figure 3): ngtdm_Coarseness, ngtdm_Strength, glszm_ZoneVariance, and glcm_DifferenceEntropy. Their importance in the radiomic model based on the SVM algorithm was 0.592, 0.503, 0.576, and 0.606, respectively, as shown in Figure 4A. As depicted in the ROC and PR curves, AUCs in the training set were 0.744 (95% CI 0.622–0.867) and 0.72 (Figure 4B,C). ACC, SEN, SPE, PPV, NPV, and Brier scores were 0.746, 0.71, 0.775, 0.71, 0.775, and 0.206, respectively. The calibration curve indicated good agreement between prediction and true value (*p* > 0.05, Figure 4D), and DCA shows the high clinical utility of the radiomic model (Figure 4E). As validation, the AUCs of ROC and PR curves were 0.733 (95% CI 0.535–0.931) and 0.72, respectively (Figure 4F,G). ACC, SEN, SPE, PPV, NPV, and Brier scores were 0.733, 0.692, 0.765, 0.692, 0.765, and 0.216, respectively. Also, the calibration curve and DCA were well‐fitted (Figure 4H,I). The distribution of Rad_score between Treg^high^ and Treg^low^ groups was significantly different in both training and validation sets (*p* < 0.05, Figure 4J,K).

**FIGURE 5:**
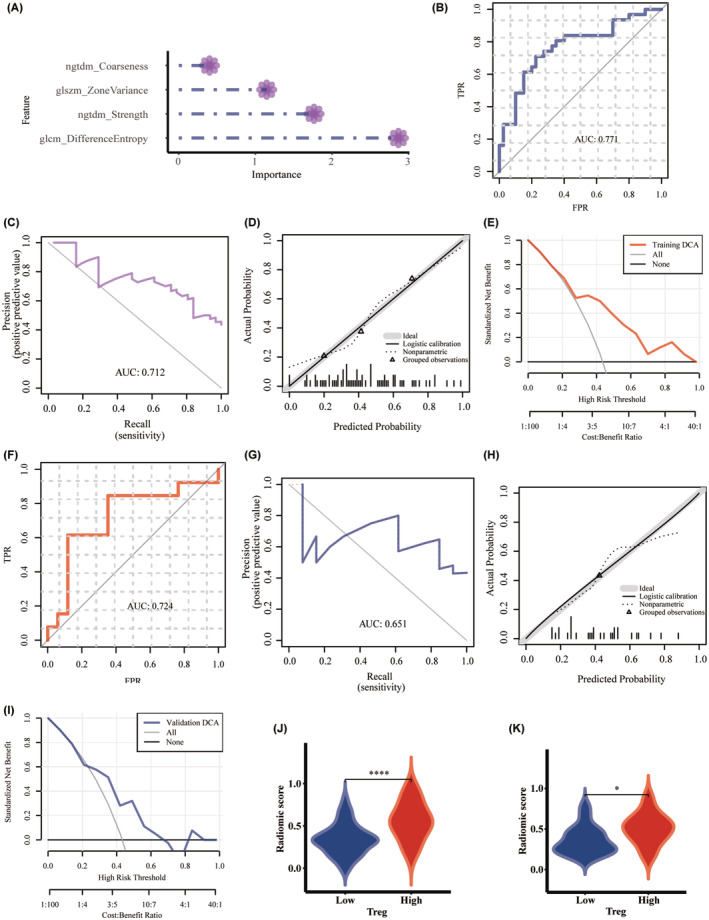
The importance of features and evaluation of the radiomic model based on logistic regression: (A) the importance of features; (B) the ROC curve, (C) the PR curve, (D) the calibration curve, and (E) DCA in the training set; (F) the ROC curve, (G) the PR curve, (H) the calibration curve and (I) DCA in the validation set; comparison of Rad_score between high and low regulatory T lymphocyte infiltrating groups in the (J) training and (K) validation sets. DCA, decision curve analysis; PR, precision‐recall; Rad_score, radiomic score; ROC, receiver operating characteristic.

Based on the LR algorithm, the importance of the radiomic model is shown in Figure 5A. The formulation of radiomic score for Treg infiltration estimation is: radiomic score = 0.234 + 0.177*original_ngtdm_Coarseness‐ 1.992*original_ngtdm_Strength+6.127*original_glszm_ZoneVariance+1.584*original_glcm_DifferenceEntropy. Again, the AUCs of ROC and PR curves in the training set were 0.771 (95% CI 0.657–0.885) and 0.712 (Figure 5B,C). ACC, SEN, SPE, PPV, NPV, and Brier scores were 0.746, 0.71, 0.775, 0.71, 0.775, and 0.192, respectively. The calibration curve indicated good agreement between prediction and true value (*p* > 0.05, Figure 5D), and DCA shows the high clinical utility of the radiomic model (Figure 5E). As validation, the AUCs of ROC and PR curves were 0.724 (95% CI 0.522–0.926) and 0.651, respectively (Figure 5F,G). ACC, SEN, SPE, PPV, NPV, and Brier scores were 0.733, 0.615, 0.647, 0.571, 0.688, and 0.211, respectively. Once again, the calibration curve and DCA performed well (Figure 5H,I). Rad_scores were also significantly different between different Treg abundance (*p* < 0.05, Figure 5J,K).

**FIGURE 6:**
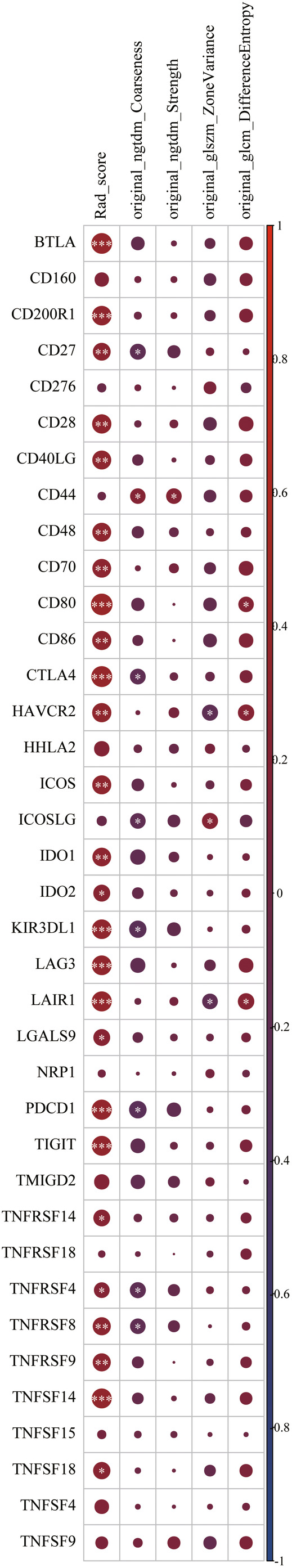
Correlation analysis between Rad_score and immune inhibitory genes. Rad_score, radiomic score; *p < 0.05, **p < 0.01, ***p < 0.001.

There was no statistical difference between the AUC values in the training and validation sets (*p* = 0.924, 0.693 for SVM and LR algorithms, respectively), indicating that the models were well generalized. For comparison between the two algorithms, the difference in ROC AUCs was not statistically significant in both training and validation sets (*p* = 0.344 and 0.779). Yet, given that the SVM model had better metrics than the LR model in the validation set, the former was selected for further correlation analysis. Rad_score was significantly correlated with the immune inhibitory genes, such as CTLA4, and PDCD1 (Figure 6).

## 4 DISCUSSION

Even though Treg only stands for 5% of CD4^+^ T lymphocytes in the blood circulation of healthy donors, their abundance increases in the tumor microenvironment (TME) of BC.^11^ In this study, we confirmed the risk exerted by Treg infiltration in TME on BC patients via landmark analysis. Given the increasing number of oncological clinical trials being done that use radiomics, we developed a radiomic score to estimate Treg infiltration in TME, which was found to be correlated to inhibitory immune genes. These results have endorsed the biological and clinical relevance of the Rad_score as a potential surrogate of Treg infiltration in clinical practice.

Nowadays, tumor‐infiltrating lymphocytes (TIL) as prognostic or predictive biomarkers are under intense evaluation. Particularly, Treg is associated with response to chemotherapy, radiotherapy, CDK4/6 inhibitor (CDKi), and immunotherapy.^12^ Indeed, Treg might play a pivotal role in BC initiation and progression, and targeting Treg in TME could restore the antitumor immune response.^13^ CDKi could reduce Treg abundance and drive the balance toward immune activation. The association between CDKi response and Treg reduction highlights the role of immune activation in solid tumor shrinkage. Combining CDKi and ICI led to total tumor regression and immunological memory.^14^ Our findings are in line with the above research. With standard medical management, over 7.7 million BC patients survived over 5 years.^15^ The landmark analysis we employed could avoid the lead time bias by interfering with factors like follow‐up time and medical history.^16^

Given the diversified therapies and the long survival time of BC patients, longitudinal evaluation is indispensable for disease control, enabling treatment adjustment.^17^ In clinical practice, monitoring mainly relies on tissue biopsy and imaging. Although the gold standard for diagnosis and treatment guidance, a biopsy is invasive, and more importantly not always feasible. Multiple sampling is unavailable in daily practice, which leads to a lack of spatial and temporal information. Considering that Treg interactions in TME have an immense impact on patients' outcomes, imaging of Treg infiltration is highly desired.^18^ Several preclinical trials for in vivo TIL imaging were applied with radiotracers or nanoparticles, whose injection is not always practical^19^ Alternatively, radiomics would allow the assessment of TME, spatial heterogeneity, and longitudinal evolution.

Thus, we present a tracer‐free imaging approach for the characterization of TME, using cell size‐based discrimination. Radiomic assessment of Treg infiltration enables oncologists to evaluate prognosis and therapeutic choice more accurately. Quantification of Treg infiltration could monitor disease progression and detect treatment response early before established tumor volumetry is detected.^20^ Also, Rad_score may serve as a screening tool before embarking on expensive immune assays like PD‐L1. What's more, it can be conveniently integrated into prognostic models with other variables (like age and TNM stages).^21^ About 27 radiomic trials have been registered on ClinicalTrials.gov.

By analyzing the linkage between radiomic features and Treg infiltration, we partially validated the radiomic algorithm,^22^ which consists of variables from the Gray Level Co‐occurrence Matrix (GLCM), Gray Level Size Zone Matrix (GLSZM), and Neighboring Gray Tone Difference Matrix (NGTDM). GLCM mathematically assesses the structural properties of images, including complexity and homogeneity. Its entropy parameters, applied in our Rad_score, have been proven to be informative of tissue structural degradation.^23^ Given the high level of structural changes caused by BC invasion, the association of Treg infiltration with GLCM entropy parameters is rationalized.

Radiomics is a relatively novel approach to learning BC complex biology, obtaining a snapshot of the underlying biology within VOI. Its high resolution promises insights for precise diagnosis, and tailored therapeutic options, and helps to understand the flow of information underlying BC, which may lead to a crucial change in clinical research.^9^ According to the correlation analysis conducted by this study, Rad_score is in association with immune genes (CD28, CTLA4, ICOS, IDO1, LAG3, PDCD1, TIGIT, and TNFSF14). This in turn explains the potential mechanism of our radiomic model to predict Treg infiltration in TME.

There are several challenges in radiomic study. Access to high‐quality data and large cohorts is quite difficult to acquire, leading to implicit biases. While the heterogeneous data retrieved from TCGA enabled us generalizable conclusions, variability in imaging acquisition parameters might be a source of the noise. Also, legal and ethical issues are required to be solved before radiomics expands to its full potential in clinical practice. Last but not least, whether Treg is causally involved in BC development, invasion, and metastasis cannot be proved from the present descriptive study.

## 5 CONCLUSION

Treg infiltration would be highly informative in the era of immunotherapy. Radiomics is a novel platform to evaluate Treg abundance in TME and develop non‐invasive biomarkers in immunotherapy. Rad_score we formulated is a cost‐effective tool for estimating Treg infiltration.

## AUTHOR CONTRIBUTIONS

**Wenying Jiang:** Writing – original draft (lead); writing – review and editing (equal). **Ruoxi Wu:** Data curation (lead); visualization (lead). **Tao Yang:** Data curation (equal); visualization (lead). **Shengnan Yu:** Methodology (lead). **Wei Xing:** Conceptualization (lead); project administration (lead); resources (lead).

## FUNDING INFORMATION

National Natural Science Foundation of China (82171901).

## CONFLICT OF INTEREST STATEMENT

The authors declare no conflicts of interest.

## ETHICS STATEMENT

The study was performed in accordance with the ethical standards as laid down in the 1964 Declaration of Helsinki and its later amendments or comparable ethical standards. No ethical review was required for the collection of de‐identified data in TCGA and TCIA.

## INFORMED CONSENT

No informed consent was required for the collection of de‐identified data in TCGA and TCIA.

## Supporting information


Figure S1.

Figure S2.

Figure S3.

## Data Availability

The datasets generated during and/or analyzed during the current study are available from the corresponding author upon reasonable request. Requests for sharing the code should be submitted to the corresponding author for consideration.
