# Supplementary figures and images for: Profiling regulatory T lymphocytes within the tumor microenvironment of breast cancer via radiomics
Source: Cancer Med. 2023 Dec 11;12(24):21861–72. doi: 10.1002/cam4.6757 (PMC10757114; doi:10.1002/cam4.6757)

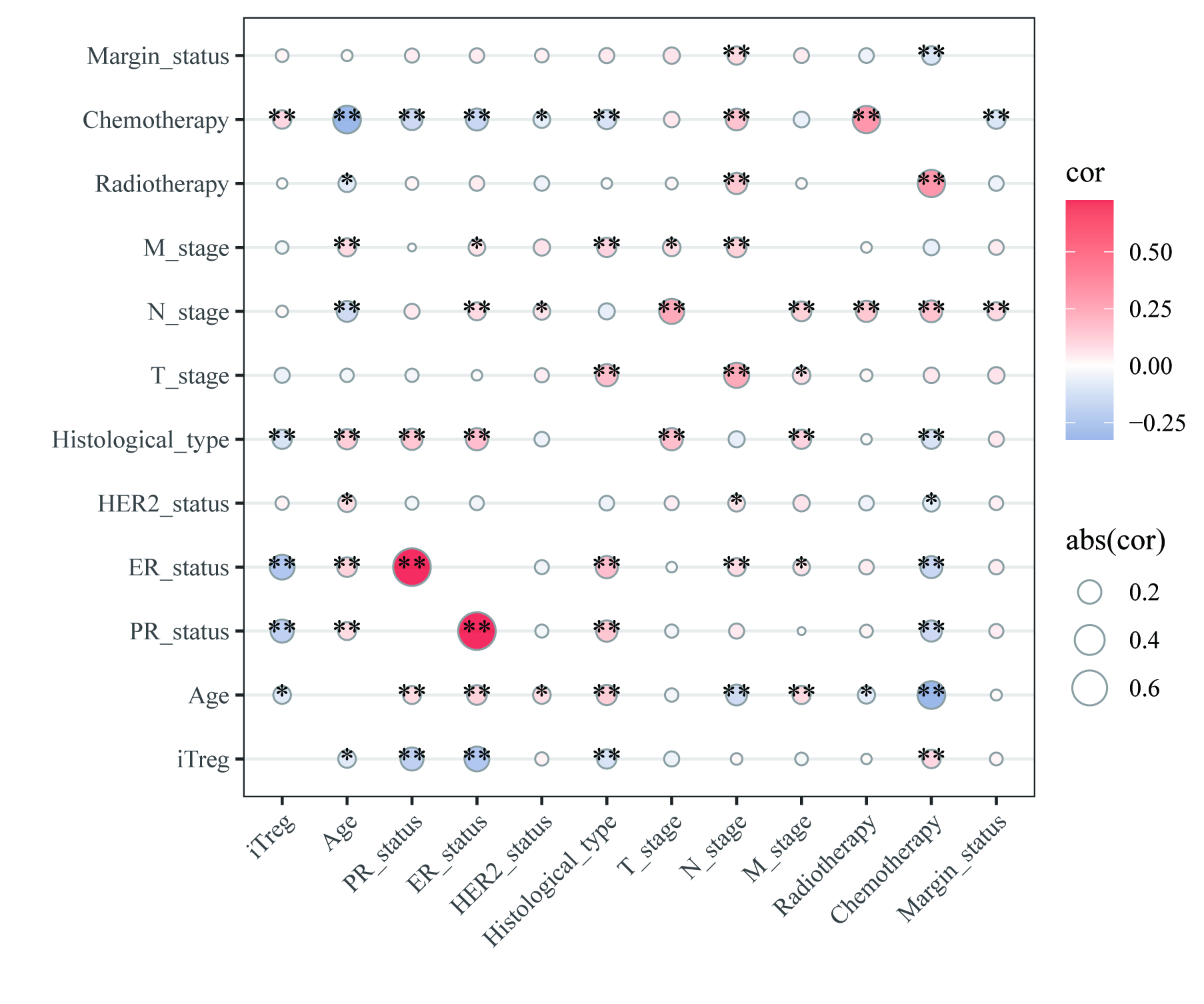

Supplement: Supplementary file 1 — Figure S1. [file CAM4-12-21861-s002.tif]

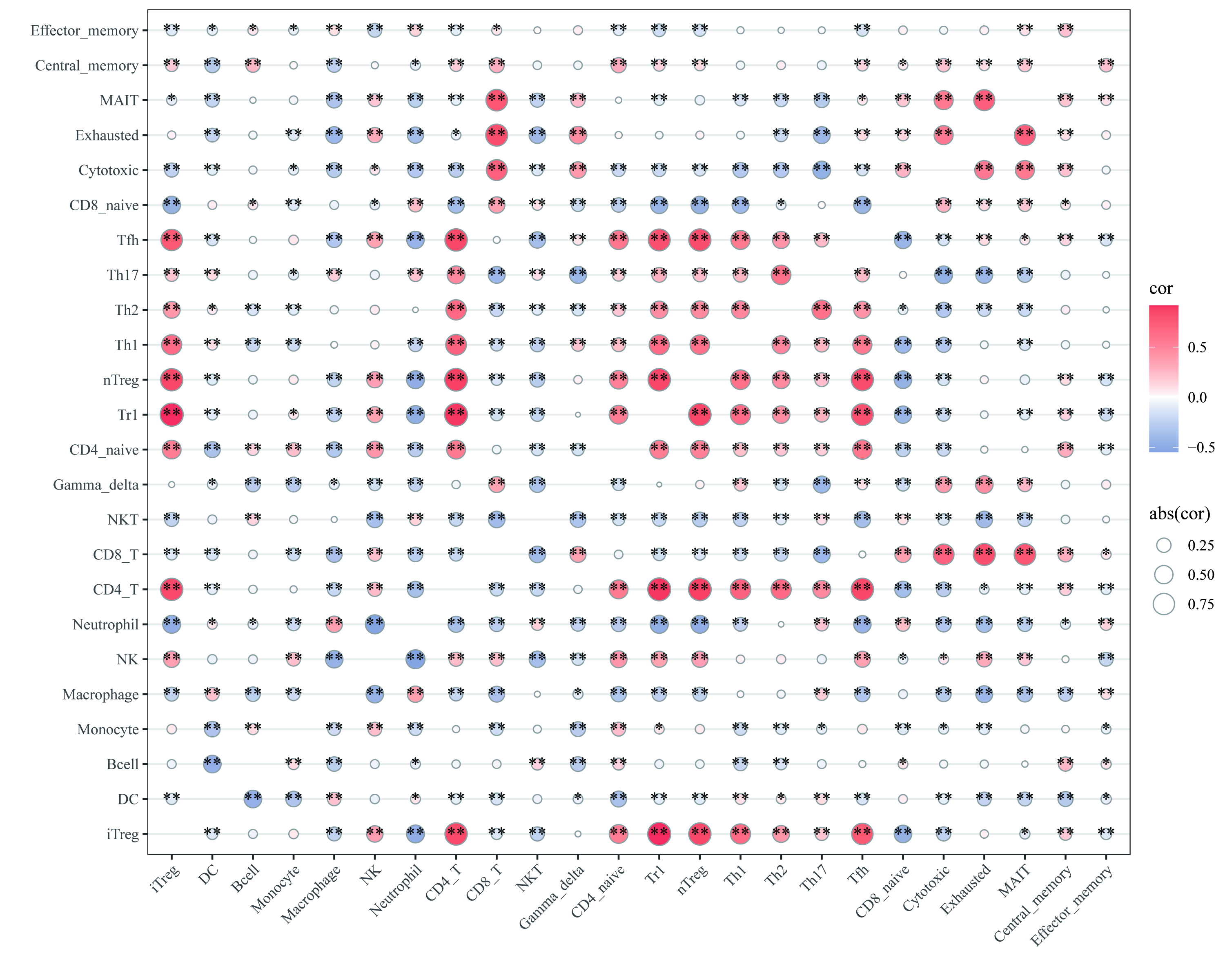

Supplement: Supplementary file 2 — Figure S2. [file CAM4-12-21861-s001.tif]

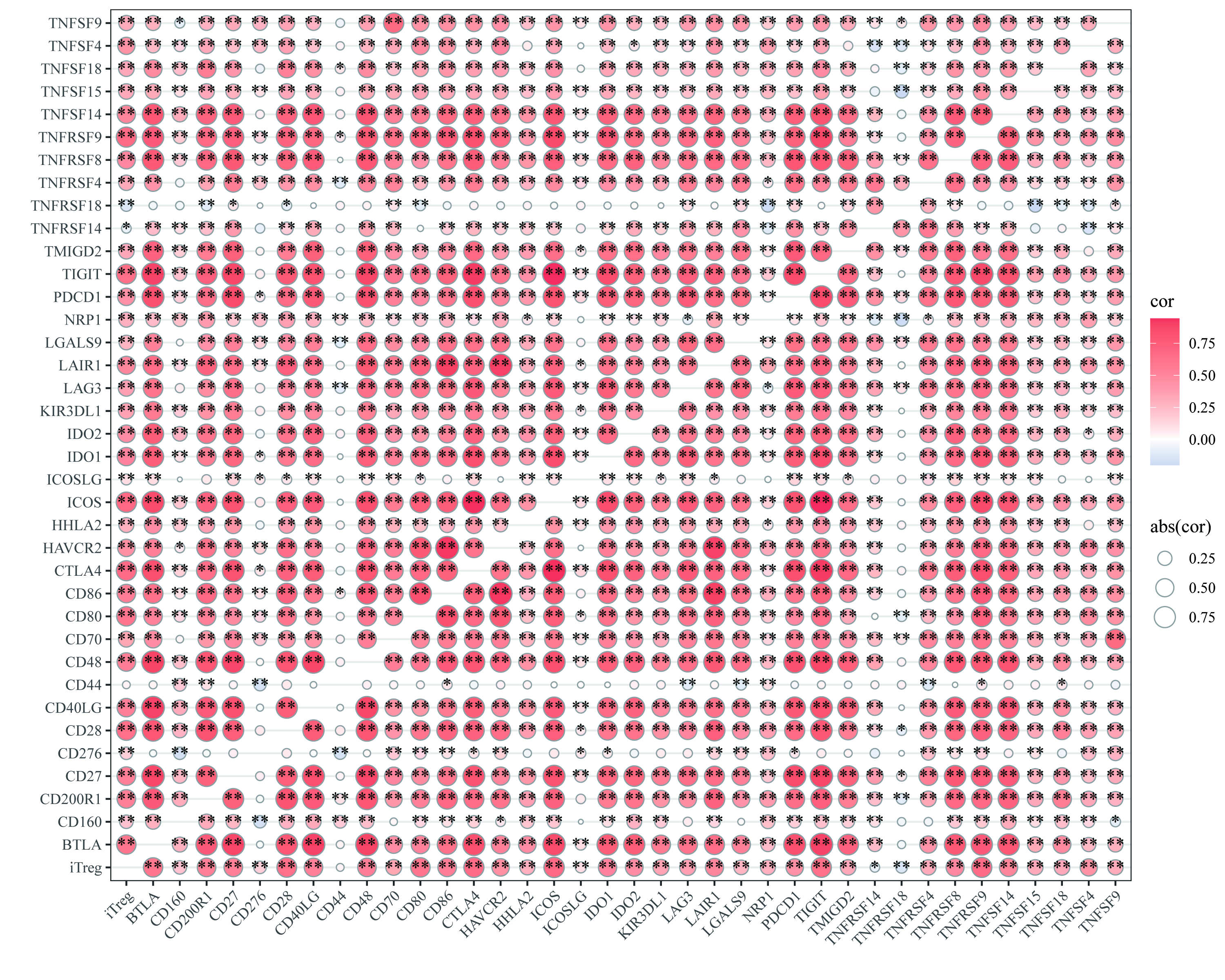

Supplement: Supplementary file 3 — Figure S3. [file CAM4-12-21861-s003.tif]
